# Joint Power Allocation and Hybrid Beamforming for Cell-Free mmWave Multiple-Input Multiple-Output with Statistical Channel State Information

**DOI:** 10.3390/s24196276

**Published:** 2024-09-27

**Authors:** Jiawei Bai, Guangying Wang, Ming Wang, Jinjin Zhu

**Affiliations:** 1 College of Electronic and Information Engineering, Nanjing University of Aeronautics and Astronautics, Nanjing 210016, China; bjw1220113@163.com (J.B.); jinjin_zhu@nuaa.edu.cn (J.Z.); 2 School of Modern Posts, Nanjing University of Posts and Telecommunications, Nanjing 210023, China; 3 School of Cyber Science and Engineering, Southeast University, Nanjing 211189, China; 220224846@seu.edu.cn

**Keywords:** cell-free MIMO, mmWave communication, sum-rate, power allocation, hybrid beamforming

## Abstract

Cell-free millimeter wave (mmWave) multiple-input multiple-output (MIMO) can effectively overcome the shadow fading effect and provide macro gain to boost the throughput of communication networks. Nevertheless, the majority of the existing studies have overlooked the user-centric characteristics and practical fronthaul capacity limitations. To solve these practical problems, we introduce a resource allocation scheme using statistical channel state information (CSI) for uplink user-centric cell-free mmWave MIMO system. The hybrid beamforming (HBF) architecture is deployed at each access point (AP), while the central processing unit (CPU) only combines the received signals by the large-scale fading decoding (LSFD) method. We further frame the issue of maximizing sum-rate subject to the fronthaul capacity constraint and minimum rate constraint. Based on the alternating optimization (AO) and fractional programming method, we present an algorithm aimed at optimizing the users’ transmit power for the power allocation (PA) subproblem. Then, an algorithm relying on the majorization–minimization (MM) method is given for the HBF subproblem, which jointly optimizes the HBF and the LSFD coefficients.

## 1. Introduction

In the future, the 6G networks need to have higher performance than 5G, supporting user rates ranging from Gbps to tens of Gbps and peak rates ranging from hundreds to even Tbps per hour, which puts forward requirements for new physical layer technology. As a pivotal technology candidate for future communication, the extremely large-scale MIMO technology is crucial for supporting the ultra-high peak rate, super-high spectral efficiency, massive connections, and extremely fast response [1,2].

In practice, larger-scale antenna systems have higher requirements for the integration of antennas in limited space. As an implementation scheme, the distributed large-scale MIMO system, also referred to as the cell-free MIMO system, is able to substantially enhance spectral efficiency and effectively expand coverage by deploying distributed RF links and antennas in a wider geographical range [3,4].

### 1.1. Related Works and Motivation

Significant research efforts have been dedicated to cell-free MIMO technologies. In [5], the team introduced an innovative, low-complexity resource allocation algorithm designed to enhance the energy efficiency using a zero forcing precoder. In [6], a short-term power constrained distributed conjugate BF method is employed to boost the capacity in multicast systems. The fronthaul is an important practical problem in the cell-free MIMO system. Various performance indicators of the system are directly affected by the fronthaul transmission of channel state information and user data. At present, some of the literature has studied the fronthaul problem. In [7], the authors compared the estimate-then-quantize and quantize-then-estimate schemes at APs and analyzed the impact of both schemes on the uplink throughput under zero forcing detection. The optimization of uplink rate and energy efficiency under limited fronthaul capacity were investigated in [8,9], respectively. In [10], the authors further combine non-orthogonal multiple access (NOMA) technology in the above scenario to optimize the worst-case rate. The work [11] develops an energy-efficient resource allocation scheme for a distributed multicell wireless power transfer enabled massive MIMO–NOMA network, which combines the user–AP connection method with the joint optimization of power control, time allocation, antenna selection, and subcarrier assignment. Unlike the aforementioned work where each user can only connect to a single AP, we consider a user-centric cell-free network in which each user can be served cooperatively by several surrounding APs, and signal processing is centrally managed by a CPU to eliminate interference between users. Meanwhile, the above work only considers the signal processing scheme in the low-frequency band, and the future communication technology will be more based on high frequency bands to improve the throughput.

The mmWave frequency band offers extensive spectral resources, effectively improving the system capacity [12]. The work [13] explored energy-efficient hybrid beamforming strategies for a satellite–terrestrial integrated network, where a multi-beam satellite system and a cellular system share the mmWave spectrum. In [14], the authors proposed novel self-powered absorptive reconfigurable intelligent surfaces to protect the satellite–terrestrial integrated networks. Specifically, in [15], the authors studied the propagation characteristics of cellular millimeter waves in urban and suburban environments. In [16], the authors introduced an advanced HBF algorithm using both analog and digital domain beamforming, while also showcasing the millimeter wave protocol, which includes a real-time baseband modem, millimeter wave radio frequency circuits, and other related software. In [17], the authors studied two millimeter wave system architectures, including fully connected structures and subarray structures, and introduced an optimal PA and HBF design algorithm derived from the binary method under both structures.

Cell-free mmWave MIMO technology has garnered considerable interest in recent years. The integration of cell-free MIMO technology with mmWave communication technology is able to offer satisfactory services to all users and solve the problems of blocking and low coverage by utilizing the macro diversity. In [18], the authors developed a hybrid precoding algorithm aimed at maximizing the weighted sum-rate. Two HBF schemes, decentralized HBF and semi-centralized HBF, were proposed in [19]. For the first mentioned, each AP independently generates an HBF matrix, while in the latter, the analog BF matrix is designed by the CPU. In [20], the analog precoding is first designed based on statistical CSI, and then the compression matrix and digital precoding are designed according to the equivalent instantaneous CSI. In [21], a max–min power allocation strategy is given to guarantee a high standard of user experience. In [22], the authors studied the downlink precoding of systems under low capacity fronthaul link. In [23], a novel dynamic subarray was introduced to enhance the global energy efficiency. A novel model was developed in [24], in which APs transmit data to a CPU using high frequency wireless links instead of wired links, and the APs serve users over a low-frequency. In [25], the author investigated the design of hybrid beamformer in multicast, unicast, and  broadcast scenarios.

Nevertheless, the aforementioned works rely on ideal instantaneous CSI, but in practical, real-time feedback of CSI in multi-user and multi-AP scenarios faces significant difficulties. Meanwhile, the HBF structure results in that the baseband channel estimator can only obtain low dimensional equivalent CSI through a few RF links, and estimating the complete channel matrix is challenging. Therefore, the minimum mean square error and least squares methods based on uplink pilots in the low frequency range are difficult to apply to millimeter wave MIMO systems. Based on this, an open-loop channel estimate method was proposed via compressed sensing techniques in [26]; however, such algorithms suffer from high complexity in practice. A mmWave channel estimator was further developed in [27], and the proposed algorithm initially utilizes frequency tone to determine the dominant angle of arrival (AoA) to design analog BF. Subsequently, the equivalent channel is used to develop a digital BF. However, this method cannot jointly design analog and digital beamforming, resulting in some performance losses. Meanwhile, several studies have explored specific aspects of power allocation and user association strategies. The work [28] examined a millimeter-wave downlink communication system supported by multiple reconfigurable intelligent surfaces, focusing on optimizing passive beamforming, power allocation, and user association to maximize the sum-rate. In [29], the authors addressed the joint problem of power allocation and user association in multi-cell massive MIMO networks, offering a solution with low computational complexity. Ref. [30] introduces a power allocation algorithm utilizing deep Q-learning to optimize both energy efficiency and throughput in 5G networks. In [31], the authors investigated the joint problem of decoupled uplink–downlink association and trajectory design in full-duplex multi-UAV networks. However, the above research did not consider the characteristics of user-centricity and limited fronthaul capacity in cell-free MIMO systems, which is discussed in detail in this paper. Table 1 compares our contributions to existing literature.

Therefore, the robust allocation strategy utilizing statistical CSI is more practical in the cell-free mmWave MIMO system, and since the line-of-sight (LOS) path of the mmWave channel occupies the main component, the optimization design based on statistical CSI will not cause significant system performance loss. This inspired us to study the robust resource allocation scheme based on statistical CSI and consider the user-centric characteristics and practical fronthaul capacity limitations in the cell-free mmWave MIMO system that most works have not explored yet.

### 1.2. Main Contributions

This paper investigates the robust resource allocation strategy relying on statistical CSI for user-centric cell-free mmWave MIMO system considering limited fronthaul capacity. The current work makes the following key contributions:

- Decentralized Cell-Free mmWave MIMO Architecture: We focus on a decentralized cell-free mmWave MIMO architecture where a HBF structure is deployed at each AP. The received signals are processed locally before being sent to the CPU. The CPU performs the weighting combination using the LSFD method, which is applied here for the first time in cell-free mmWave MIMO systems.
- Novel AP–User Association Strategy: We introduce a novel AP–user association strategy that leverages channel covariance. After pairing users with APs, we formulate a sum-rate maximization problem based on statistical CSI, subject to constraints on fronthaul capacity and minimum rate.
- Efficient Resource Allocation Scheme: We present an efficient resource allocation scheme designed to address the problem of multiple variable coupling. The experimental results show that the proposed strategy achieves a sum-rate comparable to that of benchmark schemes and that employing the LSFD method at the CPU significantly enhances the system performance.

Notations: ${\left(\cdot \right)}^{†}$ denotes the matrix generalized inversion. The complex Gaussian distribution with variance $\sigma^{2}$ and mean μ is shown as $\mathrm{CN}(\mu ,\sigma^{2})$. ${\left[\cdot \right]}_{i,j}$ is the element of the i-th row and j-th column of the matrix. *∡* indicates the eigenvector associated with the largest eigenvalue of the matrix. $C^{A\times B}$ indicates the complex matrices of dimension A×B. |·| is the absolute value or modulus of a complex number. ∥·∥ indicates the norm. E{·} indicates the average value of a variable.

## 2. System Model

**Figure 1:**
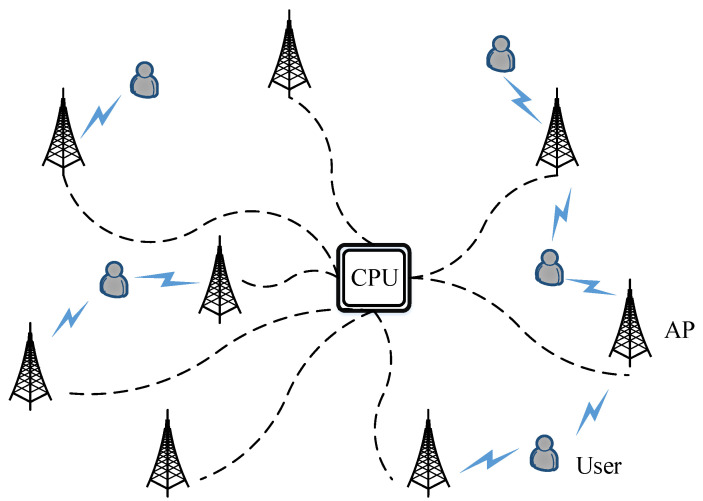
Cell-free mmWave MIMO system.

**Figure 2:**
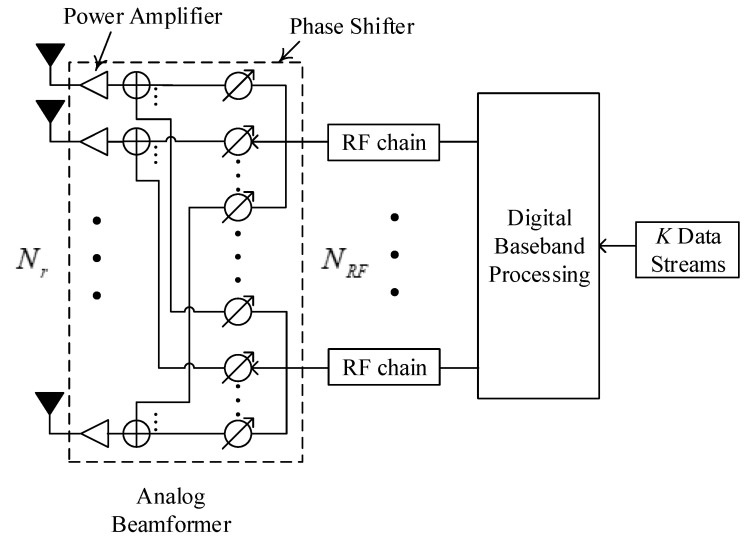
Hybrid beamforming structure at each AP.

This paper investigates a user-centric cell-free mmWave MIMO system that consists of *K* single-antenna users and *M* APs, each fitted with $N_{r}$ antennas and $N_{RF}$ RF links, and they are randomly placed within a region with a length of D. A fully connected configuration is adopted where all RF chains are linked to every antenna component employing $N_{r}$ phase shifters. It is generally assumed that $N_{r}\geq N_{RF}$ to reduce system power consumption, and $MN_{RF}\geq K$ to provide services for each user. Furthermore, we considered a user-centric architecture, where each user is simply supported by a part of the APs. The system illustration is shown in Figure 1, and the hybrid beamforming structure is shown in Figure 2.

### 2.1. Channel Model

Owing to its short wavelength, mmWave propagation has very substantial path loss and a sparse-scattering multipath effect, which makes most channel models suitable for sub-6 GHz systems no longer accurate in millimeter wave communication. Considering these characteristics, the clustered statistical mmWave MIMO channel model proposed in [32,33] is used in this paper. Specifically, the propagation environment between each AP and user is composed of $N^{\mathrm{cl}}$ scattering clusters, and each cluster has $N_{i}^{\mathrm{ray}}\left(i=1,\ldots ,N^{\mathrm{cl}}\right)$ propagation paths, and possible LOS components, where $N^{\mathrm{cl}}∼$ max{Poisson(λ),1}, with λ=1.9 a suggested value at 73 GHz, while $N_{i}^{\mathrm{ray}}$ is modeled as a uniform random integer in the range [1, 30]. The channel between the k-th user and the m-th AP is characterized as

(1) $$\begin{array}{cc} \; & h_{m,k}=h_{m,k}^{\mathrm{los}}+h_{m,k}^{\mathrm{nlos}}\\ \; & =I^{\mathrm{los}}\left(d_{m,k}^{\mathrm{los}}\right)\times \sqrt{N_{r}L\left(d_{m,k}^{\mathrm{los}}\right)}e^{j\omega_{m,k}^{\mathrm{ini}}}a\left(\varphi_{m,k}^{\mathrm{los}},\phi_{m,k}^{\mathrm{los}}\right)+\eta_{m,k}\\ \; & \times \sum_{i=1}^{N_{m,k}^{\mathrm{cl}}}\sum_{j=1}^{N_{m,k,i}^{\mathrm{ray}}}\alpha_{m,k,i,j}\sqrt{N_{r}L\left(d_{m,k,i,j}^{\mathrm{nlos}}\right)}a\left(\varphi_{m,k,i,j}^{\mathrm{nlos}}\phi_{m,k,i,j}^{\mathrm{nlos}}\right) \end{array}$$

where $h_{m,k}^{\mathrm{los}}$ and $h_{m,k}^{\mathrm{nlos}}$ denote LOS and NLOS components between the k-th user and the m-th AP, respectively. $d_{m,k}^{\mathrm{los}}$ and $d_{m,k,i,j}^{\mathrm{nlos}}$ are the length of LOS and (i,j)-th NLOS path, respectively. $I^{\mathrm{los}}\left(d_{m,k}^{\mathrm{los}}\right)$ represents a random variable that denotes whether there is a LOS path among APs and users. Defining $p^{\prime }$ as the probability of $I^{\mathrm{los}}\left(d_{m,k}^{\mathrm{los}}\right)=1$, we have $p^{\prime }=\min\left(20/d_{m,k}^{\mathrm{los}},1\right)\left(1-e^{d_{m,k}^{\mathrm{los}}/39}\right)+e^{-d_{m,k}^{\mathrm{los}}/39}$. $\omega_{m,k}^{\mathrm{ini}}∼U\left(0,2\pi \right)$ and $L(d_{m,k}^{\mathrm{los}})$ denotes the complex path gain phase and fading coefficient of the LOS path, while $\alpha_{m,k,i,j}$ and $L(d_{m,k,i,j}^{\mathrm{nlos}})$ denotes the complex gain and fading coefficient related to the (i,j)-th NLOS path, respectively, where $\alpha_{i,j}∼CN\left(0,\sigma_{\alpha ,i}^{2}\right)$ and $\sigma_{\alpha ,i}^{2}=1$. $\eta_{m,k}=\sqrt{1/\sum_{i=1}^{N_{m,k}^{\mathrm{cl}}}N_{m,k,i}^{\mathrm{ray}}}$ is a normalization factor, and a(φ,ϕ) denotes the array response vector, in which φ is the azimuth, and ϕ is the elevation. The uniform planar antenna array is adopted in each AP, thus, a(φ,ϕ) can be modeled as $a\left(\varphi ,\phi \right)=\frac{1}{\sqrt{YZ}}{\left[1,\ldots ,e^{-jk\tilde{d}\left(\left(Y-1\right)\sin\varphi \sin\phi +\left(Z-1\right)\cos\phi \right)}\right]}^{T}$, where $\tilde{d}$ is the antenna spacing, k=2π/λ and λ is the wavelength, *Y* and *Z* indicate the number of antennas on the receiver’s horizontal and vertical axes, respectively.

The large-scale fading L(d) is expressed in dB as [32]

(2) $$L\left(d\right)=-10z\left(1-l+\frac{lc}{\lambda f_{0}}\right)\mathrm{lg}\left(d\right)-20\mathrm{lg}\left(\frac{4\pi }{\lambda }\right)-X_{\sigma }$$

in which $X_{\sigma }$ represents the shadow fading coefficient that follows a logarithmic Gaussian distribution, *z* indicates the path loss coefficient, *l* refers to the system factor, and $f_{0}$ denotes a reference frequency.

However, even in time division duplex setups that take advantage of channel reciprocity, it is challenging for hybrid structures to obtain the complete channel, as the baseband estimator is able to access low dimensional precombined channels via limited RF chains. A reasonable alternative is the long-term channel statistics. A novel technique for estimating channel covariance using compressive sensing methods is proposed in [12], where the channel covariance is measured by the APs and transmitted to the CPU for global resource allocation scheme design. Compared to instantaneous channel information, the spatial channel covariance varies over a longer time scale, making estimation easier and allocating more available timeslots for useful data transmission. The effect of estimation errors on the effectiveness of our design is not addressed in this paper and will be explored in the future. The association metric between k-th user and m-th AP is

(3) $$\begin{array}{cc} metric_{m,k} & =E{∥h_{m,k}∥}^{2}\\ \; & =\mathrm{tr}\left(R_{m,k}\right) \end{array}$$

where tr(·) represents the trace of a matrix, and $R_{m,k}=E\left\{h_{m,k}h_{m,k}^{H}\right\}=I^{\mathrm{los}}\left(d_{m,k}^{\mathrm{los}}\right)\times N_{r}L\left(d_{m,k}^{\mathrm{los}}\right)a_{m,k}^{\mathrm{los}}{\left(a_{m,k}^{\mathrm{los}}\right)}^{H}+\eta_{m,k}^{2}\times \sum_{i=1}^{N_{m,k}^{\mathrm{cl}}}\sum_{j=1}^{N_{m,k,i}^{\mathrm{ray}}}N_{r}L\left(d_{m,k,i,j}^{\mathrm{nlos}}\right)a_{m,k,i,j}^{\mathrm{nlos}}{\left(a_{m,k,i,j}^{\mathrm{nlos}}\right)}^{H}$ refers to the channel covariance matrix among the k-th user and m-th AP [21].

### 2.2. Uplink Data Transmission

Over the period of uplink communication, we represent the transmit power and the symbol of the k-th user as $p_{k}$ and $x_{k}∼\mathrm{CN}\left(0,1\right)$, respectively. The received signal at the m-th AP is

(4) $$y_{m}=\sum_{k=1}^{K}h_{m,k}\sqrt{p_{k}}x_{k}+n_{m}$$

in which $n_{m}∼\mathrm{CN}\left(0,\sigma^{2}\right)$ is the additive white Gaussian noise (AWGN). Define $F_{RF,m}\in C^{N_{r}\times N_{RF}}$ as the analog BF and $f_{BB,m,k}\in C^{N_{RF}\times 1}$ as digital BF at the m-th AP.

We further define that $M_{k}$ is the number of APs serving the k-th user, while $K_{m}$ is the number of users supported by the m-th AP, and $u_{m,k}\in \left\{0,1\right\}$ is the AP–user association indicator. When $u_{m,k}=1$, it indicates that the m-th AP detects the uplink data of the k-th user. It is worth noting that under the same time-frequency resources, each AP can serve no more than $N_{RF}$ users simultaneously, so $\sum_{k=1}^{K}u_{m,k}=K_{m}\leq N_{RF}$ must be met. In situations where a large number of users need to be served by the communication network, the AP can leverage orthogonal frequency division multiple access (OFDMA) technology to allocate distinct subcarriers to individual users [34]. The total number of users an AP can support is determined by its number of RF links and available subcarriers. In this paper, we focus on the scenario where users move at low speeds, then they can be viewed as static over several coherent time periods, ensuring the feasibility of the proposed AP–user association strategy.

To adapt to the limited-capacity fronthaul link, one effective strategy is to employ quantization for signal transmission between the APs and the CPU. In practice, this quantization is achieved through the use of low-resolution analog-to-digital converters (ADCs) and digital-to-analog converters (DACs) installed on the APs. Alternatively, another approach involves utilizing codebook-based fronthaul compression, which is designed based on rate-distortion theory [22,35]. Common compression strategies include the single-user compression and Wyner–Ziv coding. In this paper, we adopt the single-user compression approach to achieve signal transmission between APs and the CPU. Specifically, the detected signal $f_{BB,m,k}^{H}F_{RF,m}^{H}y_{m}$ is mapped to a codeword, transmitted in bits through the fronthaul link, and then demapped to ${\tilde{y}}_{m,k}$ at the CPU. The detected signal for the k-th user from the m-th AP to the CPU is

(5) $$\begin{array}{cc} {\tilde{y}}_{m,k}=u_{m,k}(f_{BB,m,k}^{H} & F_{RF,m}^{H}\sum_{i=1}^{K}h_{m,i}\sqrt{p_{i}}x_{i}\\ \; & +f_{BB,m,k}^{H}F_{RF,m}^{H}n_{m}+q_{m,k}) \end{array}$$

where $q_{m,k}∼\mathrm{CN}\left(0,Q_{m,k}\right)$ denotes quantization noise, representing the distortion from the compression of signal in the fronthaul link of $C_{m}$ bps/Hz. $C_{m}$ indicates the maximum fronthaul capacity at the m-th AP. A key parameter in fronthaul compression design is the level of $q_{m,k}$ introduced by the compression operation, which is independent of $y_{m}$, and $Q_{m,k}>0$ determines the shape of the codebook. The existence of a codebook is guaranteed by the information theoretic argument proposed in [22,36] as long as the codebook size is smaller than $2^{C_{m}}$, which defines the feasible set of $Q_{m,k}$ as the ones that satisfy

(6) $$\begin{array}{cc} \; & c_{m}\left(p_{k},Q_{m,k},f_{BB,m,k},F_{RF,m}\right)=\\ \; & \sum_{k=1}^{K}u_{m,k}\log_{2}\left(1+\frac{\Lambda_{m,k}+\Xi_{m,k}}{Q_{m,k}}\right)\leq C_{m},\forall m\in M \end{array}$$

in which $\Xi_{m,k}=f_{BB,m,k}^{H}F_{RF,m}^{H}F_{RF,m}f_{BB,m,k}\sigma^{2}$ represents the power of the additive Gaussian white noise at receiver, and $\Lambda_{m,k}=\sum_{i=1}^{K}p_{i}f_{BB,m,k}^{H}F_{RF,m}^{H}h_{m,i}h_{m,i}^{H}F_{RF,m}f_{BB,m,k}$ represents the power of the desired signal. The AP set serving the k-th user is defined as $M_{k}$, then the detected signal after the LSFD at CPU is

(7) $$\begin{array}{cc} {\tilde{x}}_{k}= & \sum_{m=1}^{M}u_{m,k}a_{m,k}f_{BB,m,k}^{H}F_{RF,m}^{H}\sum_{i=1}^{K}h_{m,i}\sqrt{p_{i}}x_{i}\\ \; & +u_{m,k}a_{m,k}f_{BB,m,k}^{H}F_{RF,m}^{H}n_{m}+u_{m,k}a_{m,k}q_{m,k} \end{array}$$

where $a_{m,k}$ is the LSFD coefficients [37]. We first design $u_{m,k}$ based on statistical CSI, to allocate $M_{k}$ APs to serve the k-th user relying on the ordering of channel gains, and each AP can simultaneously serve no more than $N_{RF}$ users. Note that in the above association strategy, only some APs need to provide services to their users, which can cut down on active APs, thus cutting down system energy usage. The AP-user association algorithm (Algorithm 1) is given as follows:
**Algorithm 1** AP-user association algorithm1:**Initialize:** M={1,⋯,M}, K={1,⋯,K}, $M_{k}=⌀$, $K_{m}=⌀$, ∀m∈M, ∀k∈K2:**while** K≠⌀**do**3:   find $\left\{m^{*},k^{*}\right\}=\arg\;\max\;metric_{m,k}$4:   then $M_{k}=M_{k}\cup m^{*}$, $K_{m}=K_{m}\cup k^{*}$, $u_{m^{*},k^{*}}=1$5:   **if** $|M_{k}|=M_{k}$ **then**6:     $K=K\setminus k^{*}$7:   **end if**8:   **if** $|K_{m}|=N_{RF}$ **then**9:     $M=M\setminus m^{*}$10:   **end if**11:**end while**

### 2.3. Optimization Problem Formulation

Upon determining $u_{m,k}$, we express the k-th user’s signal to interference plus noise ratio (SINR) as

(8) $$\gamma_{k}=\frac{DS_{k}}{IUI_{k}+NI_{k}+QI_{k}}$$

where

(9) $$\begin{array}{cc} \; & DS_{k}=p_{k}{\left|\sum_{m\in M_{k}}a_{m,k}f_{BB,m,k}^{H}F_{RF,m}^{H}h_{m,k}\right|}^{2}\\ \; & IUI_{k}=\sum_{i\neq k}^{K}p_{i}{\left|\sum_{m\in M_{k}}a_{m,k}f_{BB,m,k}^{H}F_{RF,m}^{H}h_{m,i}\right|}^{2}\\ \; & NI_{k}=\sum_{m\in M_{k}}\left|\right|a_{m,k}f_{BB,m,k}^{H}F_{RF,m}^{H}{\left|\right|}_{2}^{2}\sigma^{2}\\ \; & QI_{k}=\sum_{m\in M_{k}}a_{m,k}^{2}Q_{m,k} \end{array}$$

where $DS_{k}$ indicates the strength of target signal, $IUI_{k}$ is the interference among users, $NI_{k}$ indicates the effect of AWGN, $QI_{k}$ indicates the effect of quantization noise. The sum-rate maximization issue is expressed by

(10) $$\begin{array}{cc} \max_{X}\;\; & J=\sum_{k=1}^{K}E\left\{\log_{2}\left(1+\gamma_{k}\right)\right\}\\ \;s.t.\; & C_{1}:0<p_{k}\leq P_{max,k},\forall k\in K\\ \; & C_{2}:E\left\{R_{k}\right\}\geq R_{min,k},\forall k\in K\\ \; & C_{3}:E\left\{c_{m}\right\}\leq C_{m},\forall m\in M\\ \; & C_{4}:Q_{m,k}\geq 0,\forall m\in M_{k},\forall k\in K\\ \; & C_{5}:\left|{\left[F_{RF,m}\right]}_{i,j}\right|=1/\sqrt{N_{r}},\forall m\in M\\ \; & C_{6}:{∥f_{BB,m,k}^{H}F_{RF,m}^{H}∥}_{2}^{2}=1,\forall m\in M_{k},\forall k\in K \end{array}$$

where $X=\left\{F_{RF,m},f_{BB,m,k},p_{k},Q_{m,k},a_{m,k}\right\}$ denotes the variable set. $C_{1}$ denotes maximum transmit power constraint, $C_{2}$ denotes minimum rate constraint, $C_{3}$ denotes the maximum fronthaul capacity constraint, $C_{4}$ denotes the quantization error variance constraint, $C_{5}$ denotes constant modulus constraint of analog BF, and $C_{6}$ denotes the normalization of HBF at APs. In order to handle the difficult form of matrix summation in norm, the auxiliary variables $w_{i,j}^{H}=a_{i,j}f_{BB,i,j}^{H}F_{RF,i}^{H}\in C^{1\times N_{r}}$ are first introduced and define ${\tilde{h}}_{k}={\left[h_{m_{1},k}^{H},\cdots ,h_{m_{M_{k}},k}^{H}\right]}^{H}\in C^{N_{r}M_{k}\times 1}$, ${\tilde{w}}_{k}={\left[w_{m_{1},k}^{H},\cdots ,w_{m_{M_{k}},k}^{H}\right]}^{H}\in C^{N_{r}M_{k}\times 1}$. Approximately, the achievable rate expression is [38]

(11) $$\begin{array}{cc} E\{\log_{2} & \left(1+\gamma_{k}\right)\}\approx \log_{2}\left(1+E\left\{\gamma_{k}\right\}\right)\\ \; & \approx \log_{2}\left(1+\frac{E\{DS_{k}\}}{E\{IUI_{k}+NI_{k}+QI_{k}\}}\right)≜{\hat{R}}_{k} \end{array}$$

in which

(12) $$\begin{array}{cc} \; & E\left\{DS_{k}\right\}=E\{p_{k}|{\tilde{w}}_{k}^{H}{\tilde{h}}_{k}{|}^{2}\}=p_{k}{\tilde{w}}_{k}^{H}{\tilde{R}}_{k}{\tilde{w}}_{k}\\ \; & E\left\{IUI_{k}\right\}=E\{\sum_{i\neq k}^{K}p_{i}|{\tilde{w}}_{k}^{H}{\tilde{h}}_{i}{|}^{2}\}=\sum_{i\neq k}^{K}p_{i}{\tilde{w}}_{k}^{H}{\tilde{R}}_{i}{\tilde{w}}_{k}\\ \; & E\left\{NI_{k}\right\}=E\{||{\tilde{w}}_{k}^{H}{\left|\right|}_{2}^{2}\sigma^{2}\}={\tilde{w}}_{k}^{H}{\tilde{w}}_{k}\sigma^{2}\\ \; & E\left\{QI_{k}\right\}=\sum_{m\in M_{k}}\left|\right|w_{m,k}^{H}{\left|\right|}_{2}^{2}Q_{m,k} \end{array}$$

where ${\tilde{R}}_{k}=\mathrm{diag}\left(R_{m_{1},k},\ldots ,R_{m_{M_{k}},k}\right)$. Then $C_{3}$ can be similarly approximated as

(13) $$\begin{array}{cc} \log_{2}(1+ & \frac{w_{m,k}^{H}\left(\sum_{i=1}^{K}p_{i}R_{m,i}+\sigma^{2}\right)w_{m,k}/a_{m,k}^{2}}{Q_{m,k}})\\ \; & ≜{\hat{c}}_{m,k}\leq C_{m}/K_{m},\forall m\in M_{k},\forall k\in K \end{array}$$

## 3. HBF Design and Power Allocation

To maximize the sum-rate, a resource allocation scheme was proposed in this section. As a result of the auxiliary variable $w_{m,k}$, the initial optimization problem (10) can be separated into two distinct subproblems as follows

(14) $$\begin{array}{cc} \left(P1\right):\;\max_{\left\{X_{1}\right\}}\; & J_{1}=\sum_{k=1}^{K}{\hat{R}}_{k}\\ \;s.t.\;\; & C_{1}:0<p_{k}\leq P_{max,k},\forall k\in K\\ \; & C_{2}:{\hat{R}}_{k}\geq R_{min,k},\forall k\in K\\ \; & C_{3}:{\hat{c}}_{m,k}\leq C_{m}/K_{m},\forall m\in M_{k},\forall k\in K\\ \; & C_{4}:Q_{m,k}\geq 0,\forall m\in M_{k},\forall k\in K \end{array}$$

(15) $$\begin{array}{cc} \left(P2\right):\;\min_{\left\{X_{2}\right\}}\; & J_{2}=\sum_{m=1}^{M}\sum_{k=1}^{K}{∥w_{m,k}-a_{m,k}F_{RF,m}f_{BB,m,k}∥}_{2}^{2}\\ \;s.t.\;\; & C_{5}:\left|{\left[F_{RF,m}\right]}_{i,j}\right|=1/\sqrt{N_{r}},\forall m\in M\\ \; & C_{6}:{∥f_{BB,m,k}^{H}F_{RF,m}^{H}∥}_{2}^{2}=1,\forall m\in M_{k},\forall k\in K \end{array}$$

where $X_{1}=\left\{p_{k},w_{m,k},Q_{m,k}\right\}$ denotes the variable set of (P1), $X_{2}=\left\{F_{RF,m},f_{BB,m,k},a_{m,k}\right\}$ denotes the variable set of (P2).

### 3.1. Power Allocation Scheme

We first solve (P1) in this subsection, which is a multiple-variable coupling problem that can be tackled using the AO algorithm [39]. During the t-th iteration, the following steps are processed:

(1) Optimize $p_{k}^{\left(t\right)}$ for fixed $\left\{w_{m,k}^{\left(t-1\right)},Q_{m,k}^{\left(t-1\right)}\right\}$.

(16) $$\begin{array}{cc} \max_{\left\{p_{k}\right\}} & J_{1.1}=\sum_{k=1}^{K}\log\left(1+\frac{p_{k}{\bar{z}}_{k,k}}{\sum_{i\neq k}^{K}p_{i}{\bar{z}}_{k,i}+{\bar{\Delta }}_{k}}\right)\\ \;s.t.\; & C_{1}:0<p_{k}\leq P_{max,k},\forall k\in K\\ \; & C_{2}:{\hat{R}}_{k}\geq R_{min,k},\forall k\in K\\ \; & C_{3}:{\hat{c}}_{m,k}\leq C_{m}/K_{m},\forall m\in M_{k},\forall k\in K \end{array}$$

where ${\bar{\Delta }}_{k}={\tilde{w}}_{k}^{H}{\tilde{w}}_{k}\sigma^{2}+\sum_{m\in M_{k}}\left|\right|w_{m,k}^{H}{\left|\right|}_{2}^{2}Q_{m,k}$, ${\bar{z}}_{k,i}={\tilde{w}}_{k}^{H}{\tilde{R}}_{i}{\tilde{w}}_{k}$. The above non-convex problem in the form of sum-log is challenging to solve directly. For unconstrained power optimization problems, the sequential convex approximation (SCA) algorithm [40] and concave convex process (CCCP) algorithm [41] can be combined with first-order Taylor approximation to find suboptimal closed-form solutions. Its low complexity feature is more conducive to practical implementation, but it is difficult to handle many practical constraints. In this paper, we use the fractional programming algorithm to approximate the original objective function and some complex constraints into convex, and then the efficient interior point method can be used to find the optimal solution within the feasible domain, which is suitable for large-scale convex optimization problems. By introducing the auxiliary variable ${\left\{\vartheta_{k}\right\}}_{k\in K}$ based on fractional programming [39,42], the objective $J_{1.1}$ is transformed into

(17) $${\bar{J}}_{1.1}=\sum_{k=1}^{K}\log\left(1+2\vartheta_{k}\sqrt{p_{k}{\bar{z}}_{k,k}}-\vartheta_{k}^{2}\left(\sum_{i\neq k}^{K}p_{i}{\bar{z}}_{k,i}+{\bar{\Delta }}_{k}\right)\right)$$

Obviously, ${\bar{J}}_{1.1}$ is a concave function about ${\left\{\vartheta_{k}\right\}}_{k\in K}$, so in the r-th iteration of fractional programming algorithm, $\vartheta_{k}^{\left(r\right)}$ can be first updated by

(18) $$\vartheta_{k}^{\left(r\right)}=\frac{\sqrt{p_{k}^{\left(r-1\right)}{\bar{z}}_{k,k}}}{\sum_{i=1}^{K}p_{i}^{\left(r-1\right)}{\bar{z}}_{k,i}+{\bar{\Delta }}_{k}}$$

Next, introduce auxiliary variables ${\left\{{\tilde{\gamma }}_{k}\right\}}_{k\in K}$, then the optimization problem of $p_{k}$ is reformulated as

(19) $$\begin{array}{cc} \max_{\left\{p_{k},{\tilde{\gamma }}_{k}\right\}} & {\tilde{J}}_{1.1}=\sum_{k=1}^{K}\log\left(1+{\tilde{\gamma }}_{k}\right)\\ s.t. & 0<p_{k}\leq P_{\max,k},\\ \; & p_{k}{\bar{z}}_{k,k}-\left(2^{R_{min,k}}-1\right)\left(\sum_{i\neq k}^{K}p_{i}{\bar{z}}_{k,i}+{\bar{\Delta }}_{k}\right)\geq 0,\\ \; & \sum_{i=1}^{K}p_{i}\kappa_{m,k,i}+\varpi_{m,k}\sigma^{2}\leq \left(2^{C_{m}/K_{m}}-1\right)Q_{m,k},\\ \; & 2\vartheta_{k}^{\left(r\right)}\sqrt{p_{k}{\bar{z}}_{k,k}}-{\left(\vartheta_{k}^{\left(r\right)}\right)}^{2}\left(\sum_{i\neq k}^{K}p_{i}{\bar{z}}_{k,i}+{\bar{\Delta }}_{k}\right)\geq {\tilde{\gamma }}_{k}. \end{array}$$

where $\kappa_{x,y,z}=w_{x,y}^{H}R_{x,z}w_{x,y}/a_{x,y}^{2}$, $\varpi_{i,j}=w_{i,j}^{H}w_{i,j}/a_{i,j}^{2}$. The problem described above is obviously convex about ${\left\{{\tilde{\gamma }}_{k},p_{k}\right\}}_{k\in K}$, which can be solved through the cvx toolbox. When ${\bar{J}}_{1.1}$ converges in the loop of fractional programming algorithm, $p_{k}^{\left(t\right)}$ can be updated by

(20) $$p_{k}^{\left(t\right)}=p_{k}^{\left(r\right)}$$

(2) Optimize $w_{m,k}^{\left(t\right)}$ for fixed $\left\{p_{k}^{\left(t\right)},Q_{m,k}^{\left(t-1\right)}\right\}$.

Observing (8), it is obvious that SINR only depends on the ratio of LSFD coefficients. Therefore, we assume that $\sum_{m\in M_{k}}a_{m,k}^{2}=1$ for convenience, then ${∥{\tilde{w}}_{k}∥}_{2}^{2}=1$ due to $\left|\right|w_{m,k}{\left|\right|}_{2}^{2}=a_{m,k}^{2}$. Furthermore, decompose the original problem into *K* distinct subproblems, and express the k-th subproblem as follows:

(21) $$\max_{\left\{{\tilde{w}}_{k}\right\}}J_{1.2}=\frac{{\tilde{w}}_{k}^{H}A_{k}{\tilde{w}}_{k}}{{\tilde{w}}_{k}^{H}B_{k}{\tilde{w}}_{k}}$$

where

(22) $$\begin{array}{cc} \; & A_{k}=p_{k}{\tilde{R}}_{k}\\ \; & B_{k}=\sum_{i\neq k}^{K}p_{i}{\tilde{R}}_{i}+I_{N_{r}M}\sigma^{2}+\mathrm{diag}\left(Q_{m_{1},k}I_{N_{r}},\ldots ,Q_{m_{M_{k}}}I_{N_{r}}\right) \end{array}$$

$J_{1.2}$ is the form of a generalized Rayleigh quotient, so there is an optimal solution, as follows:

(23) $${\tilde{w}}_{k}^{\left(t\right)}=∡\left(B_{k}^{-1}A_{k}\right)$$

(3) Optimize $Q_{m,k}^{\left(t\right)}$ for fixed $\left\{p_{k}^{\left(t\right)},w_{m,k}^{\left(t\right)}\right\}$.

Through simple mathematical transformation, $Q_{m,k}^{\left(t\right)}$ is updated as follows:

(24) $$Q_{m,k}^{\left(t\right)}=\frac{\Theta_{m,k}}{2^{C_{m}/K_{m}}-1}$$

where $\Theta_{m,k}=w_{m,k}^{H}\left(\sum_{i=1}^{K}p_{i}R_{m,i}+\sigma^{2}\right)w_{m,k}/a_{m,k}^{2}$.

### 3.2. Hybrid Beamforming Design

We solve (P2) in this subsection through the AO and MM method. (P2) can be decomposed into *M* subproblems, and the m-th subproblem is

(25) $$\begin{array}{cc} \min_{\left\{X_{2}\right\}} & \sum_{k\in K_{m}}{∥w_{m,k}-a_{m,k}F_{RF,m}f_{BB,m,k}∥}_{2}^{2}\\ \;s.t.\; & C_{5}:\left|{\left[F_{RF,m}\right]}_{i,j}\right|=1/\sqrt{N_{r}}\\ \; & C_{6}:||f_{BB,m_{i},k}^{H}F_{RF,m}^{H}{\left|\right|}_{2}^{2}=1,\forall k\in K_{m} \end{array}$$

Note that $a_{m,k}$ is just right equal to the modulus of $w_{m,k}$ due to $\left|\right|f_{BB,m,k}^{H}F_{RF,m}^{H}{\left|\right|}_{2}^{2}=1$. This is because the CPU has already optimized $a_{m,k}$ when globally optimizing $w_{m,k}$ in (22). Thus

(26) $$a_{m,k}^{*}=\left|\right|w_{m,k}{\left|\right|}_{2}$$

Then, for the given initial values $F_{RF,m}^{\left(0\right)}$ and $f_{BB,m,k}^{\left(0\right)}$, the subsequent steps are processed during the s-th iteration.

(1) Optimize $F_{RF,m}^{\left(s\right)}$ for fixed $f_{BB,m,k}^{\left(s-1\right)}$.

The MM method is utilized to resolve the problem. First let $F_{RF,m}={\left[x_{1},\ldots ,x_{N_{r}}\right]}^{H}$, in which $x_{i}$ indicates the conjugate transpose of the i-th row of $F_{RF,m}$, then the initial problem can be decomposed into $N_{r}$ subproblems as follows:

(27) minxi∑k=1K|fBB,m,kHxi−wm,k/am,ki*|2=xiHD˜xi−2RexiHd˜i+∑k=1Kwm,k/am,ki2s.t.|[xi]j|=1/Nr

where ${\tilde{d}}_{i}=\sum_{k=1}^{K}f_{BB,m,k}{\left[w_{m,k}/a_{m,k}\right]}_{i}^{*}$ and $\tilde{D}=\sum_{k=1}^{K}f_{BB,m,k}f_{BB,m,k}^{H}$. Using $x_{i}^{\left(v-1\right)}$ to stand for the value in (v−1)-th iteration of the MM algorithm, then the tight upper bound of $x_{i}^{H}\tilde{D}x_{i}$ in the v-th iteration can be expressed as xiHD˜xi≤xiHD^xi−2Re{xiHyi(v−1)}+(xi(v−1))Hyi(v−1), where $y_{i}^{\left(v-1\right)}=\left(\hat{D}-\tilde{D}\right)x_{i}^{\left(v-1\right)}$, $\hat{D}=\lambda_{\max}\left(\tilde{D}\right)I_{N_{RF}}$, while $\lambda_{\max}\left(\tilde{D}\right)$ is the maximum eigenvalue of $\tilde{D}$ [21]. In the v-th iteration, the problem (27) is approximated as

(28) maxxiRexiHy˜i(v−1)s.t.|xij|=1/Nr

where ${\tilde{y}}_{i}^{\left(v-1\right)}=y_{i}^{\left(v-1\right)}+{\tilde{d}}_{i}$. The above problems can be further divided into $N_{RF}$ subproblems, as follows:

(29) $$\begin{array}{cc} \max_{\psi_{i,j}} & \cos\left(\varphi_{i,j}^{\left(v-1\right)}-\psi_{i,j}\right)\\ \;s.t.\; & 0\leq \psi_{i,j}\leq 2\pi \end{array}$$

in which $\varphi_{i,j}^{\left(v-1\right)}$ and $\psi_{i,j}$ denotes the phase of ${\left[{\tilde{y}}_{i}^{\left(v-1\right)}\right]}_{j}$ and ${\left[x_{i}\right]}_{j}$, respectively, and it is obvious that $\psi_{i,j}^{opt}=\varphi_{i,j}^{\left(v-1\right)}$. Thus $x_{i}^{\mathrm{opt}}=\frac{1}{\sqrt{N_{r}}}{\left[\exp\left(j\psi_{i,1}^{\mathrm{opt}}\right),\ldots ,\exp\left(j\psi_{i,N_{RF}}^{\mathrm{opt}}\right)\right]}^{T}$, i.e.,

(30) $$F_{RF,m}^{\left(v\right)}={\left[x_{1}^{\mathrm{opt}},\ldots ,x_{N_{r}}^{\mathrm{opt}}\right]}^{H}$$

When the MM algorithm converges, $F_{RF,m}^{\left(s\right)}=F_{RF,m}^{\left(v\right)}$. It should be noted that in practical applications of the AO and MM algorithm, if the initial point selection is not good, it may converge to a local optimal solution. To solve this problem, we first run the AO and MM algorithms from multiple different initial points to increase the chance of finding the global optimal solution. Furthermore a randomization strategy is introduced to randomly perturb the solution during the iteration. Specifically, random points are generated during the iteration to compare with the current iteration point, which helps to escape from the local optima. Meanwhile, the successful implementation of the MM algorithm also depends on the complexity of the beamforming design. Compared with centralized MIMO architecture, cell-free MIMO architecture greatly reduces the number of antennas and RF links on each AP, ensuring the feasibility of the MM algorithm.

(2) Optimize $f_{BB,m,k}^{\left(s\right)}$ for fixed $F_{RF,m}^{\left(s\right)}$.

Divide the initial problem into $K_{m}$ subproblems, as follows:

(31) $$\min_{\left\{f_{BB,m,k}\right\}}{∥w_{m,k}-a_{m,k}F_{RF,m}f_{BB,m,k}∥}_{2}^{2}$$

The optimal solution to (31) is given by

(32) $$f_{BB,m,k}^{\left(s\right)}={\left(F_{RF,m}^{H}F_{RF,m}\right)}^{†}F_{RF,m}^{H}w_{m,k}/a_{m,k}$$

The convergence of Algorithms 2 and 3 can be assured by the proven convergence of the AO algorithm [43], fractional programming algorithm [39,42], and MM algorithm [44]. The subsequent discussion addresses the complexity of Algorithms 2 and 3. For Algorithm 2, the complexity primarily arises from Steps 9 and 10. The standard convex optimization tools (such as CVX in [45]) are utilized to address (19) in Step 9. The complexity of the interior point method is approximated as $O(I_{2}{\left(2K\right)}^{3.5}\ln\left(1/\epsilon \right))$ because of the number of real variables in (19) being 2K, where ϵ is the precision of the solution, $I_{2}$ is the iterations of the fractional programming algorithm. The complexity of the inverse calculation of $B_{k}$ is expressed as $O(K{\left(N_{r}M_{max}\right)}^{3})$ during Step 10. Thus, the complexity of Algorithm 2 is $O(I_{1}\left(I_{2}{\left(2K\right)}^{3.5}\ln\left(1/\epsilon \right)+K{\left(N_{r}M_{max}\right)}^{3}\right))$, in which $I_{1}$ is the iterations of the AO algorithm. For Algorithm 3, its complexity primarily stems from Steps 9 and 10. The complexity of solving the eigenvalues of $\tilde{D}$ in Step 9 is $O(I_{4}MN_{r}{\left(N_{RF}\right)}^{3})$, where $I_{4}$ is the iterations of the MM algorithm. In Step 10, the complexity of the inverse calculation of $F_{RF,m}^{H}F_{RF,m}$ is $O(MKN_{RF}^{3})$. The complexity of Algorithm 3 is $O(I_{3}\left(I_{4}MN_{r}{\left(N_{RF}\right)}^{3}+MKN_{RF}^{3}\right))$, where $I_{3}$ is the iterations of the AO algorithm.
**Algorithm 2** Power Allocation Algorithm for (P1)1:**Initialize:** outer iterations t=0, outer threshold $t_{max}$, outer tolerance $\epsilon_{1}>0$, $p_{k}^{\left(0\right)}$, $w_{m,k}^{\left(0\right)}$, $Q_{m,k}^{\left(0\right)}$2:**repeat**3:   t=t+14:   **initialize:** inner iterations r=0, inner threshold $r_{max}$, inner tolerance $\epsilon_{2}>0$5:   **repeat**6:     update $\vartheta_{k}^{\left(r\right)}$ according to (18)7:     update $p_{k}^{\left(r\right)}$ according to (20)8:   **until** $\left|\right|{\bar{J}}_{1.1}^{\left(r\right)}-{\bar{J}}_{1.1}^{\left(r-1\right)}\left|\right|<\epsilon_{2}$ or $r>r_{max}$9:   update $p_{k}^{\left(t\right)}=p_{k}^{\left(r\right)}$ for fixed $\left\{w_{m,k}^{\left(t-1\right)},Q_{m,k}^{\left(t-1\right)}\right\}$10: update $w_{m,k}^{\left(t\right)}$ according to (22) for fixed $\left\{p_{k}^{\left(t\right)},Q_{m,k}^{\left(t-1\right)}\right\}$11: update $Q_{m,k}^{\left(t\right)}$ according to (23) for fixed $\left\{p_{k}^{\left(t\right)},w_{m,k}^{\left(t\right)}\right\}$12:**until**$\left|\right|J_{1}^{\left(t\right)}-J_{1}^{\left(t-1\right)}\left|\right|<\epsilon_{1}$ or $t>t_{max}$

**Algorithm 3** HBF Design Algorithm for (P2)
1:**Initialize:** outer iterations s=0, outer threshold $s_{max}$, outer tolerance $\epsilon_{3}>0$, $F_{RF,m}^{\left(0\right)}$, $f_{BB,m,k}^{\left(0\right)}$2:update $a_{m,k}$ according to (25)3:
**repeat**
4:   s=s+15:   **initialize:** inner iterations v=0, inner threshold $v_{max}$, inner tolerance $\epsilon_{4}>0$6:   **repeat**7:     update $F_{RF,m}^{\left(v\right)}$ according to (30)8:   **until** $\left|\right|F_{RF,m}^{\left(v\right)}-F_{RF,m}^{\left(v-1\right)}\left|\right|<\epsilon_{3}$ or $v>v_{max}$9:   update $F_{RF,m}^{\left(s\right)}=F_{RF,m}^{\left(v\right)}$10: update $f_{BB,m,k}^{\left(s\right)}$ according to (32) for fixed $F_{RF,m}^{\left(s\right)}$11:**until**$\left|\right|J_{2}^{\left(s\right)}-J_{2}^{\left(s-1\right)}\left|\right|<\epsilon_{4}$ or $s>s_{max}$


**Table 1:** Comparing our contribution to existing literature.

	[7]	[18]	[19]	[22]	[23]	[24]	[25]	[33]	[46]	Proposed
mmWave		✓	✓	✓	✓	✓	✓	✓	✓	✓
HBF		✓	✓	✓	✓		✓	✓		✓
Uplink	✓		✓				✓		✓	✓
Fronthaul	✓			✓						✓
User centric						✓		✓	✓	✓

## 4. Simulation Results

In the following section, the Matlab R2018b was employed to simulate and analyze the performance of the cell-free mmWave MIMO system under the developed resource allocation scheme relying on statistical CSI. In general, we suppose that the minimum rate $R_{min,k}=R_{min}$, ∀k∈K, maximum transmit power $P_{max,k}=P_{max}$, ∀k∈K, maximum fronthaul capacity $C_{m}=C_{max}$, ∀m∈M, and the number of APs supporting each user $M_{k}=M_{max}$, ∀k∈K. The users and APs are randomly distributed within a region of D×D. Table 2 outlines the detailed default parameters for evaluation.

### 4.1. Convergence Behaviour

**Figure 3:**
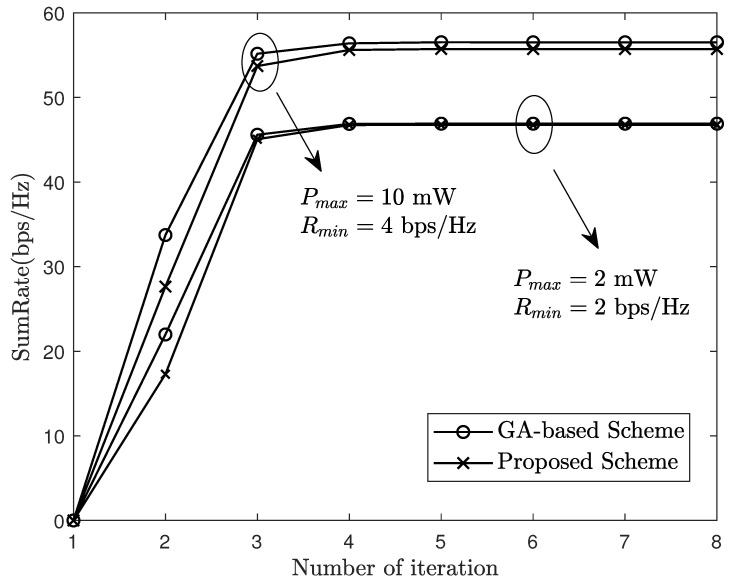
Sum-rate of system under different power allocation schemes.

Figure 3 illustrates a comparison of behavior under the genetic algorithm (GA) and the proposed scheme. As problem (16) is an optimization problem that includes minimum rate constraints and maximum fronthaul capacity constraints, the common power allocation methods, such as the SCA algorithm and CCCP algorithm, are challenging to use to address such a problem. Therefore, the penalty function method was leveraging to transfer constraint conditions to the objective function and use GA as the benchmark for comparison. It shows that with the proposed scheme, the sum-rate closely matches that achieved with the benchmark scheme based on GA. Furthermore, on the Matlab platform, the proposed scheme takes 23.18 s, while the latter takes 77.93 s, which proves the advantages of our scheme. Furthermore, Figure 3 verifies the convergence of our algorithm.

### 4.2. Performance Evaluation

**Figure 4:**
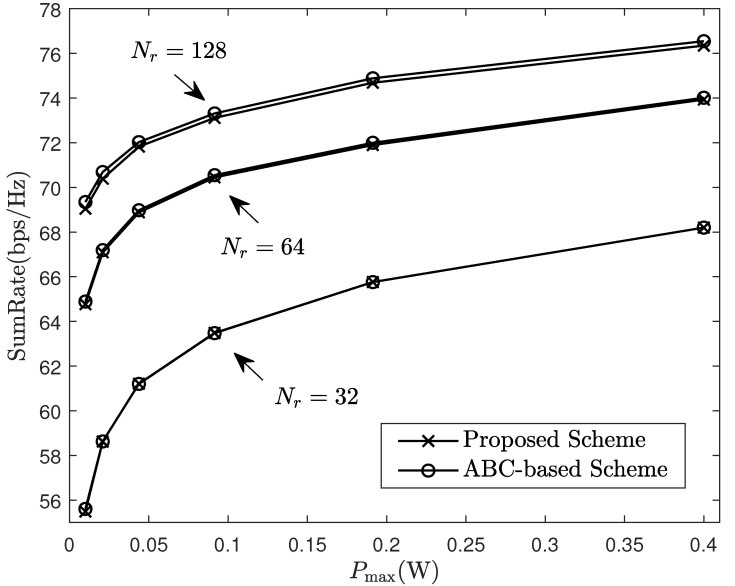
Sum-rate of system under different beamforming schemes.

Figure 4 compares the performance under different BF schemes. Owing to the constant modulus constraint in (P2), some of the existing smart algorithms, for example, particle swarm optimization and genetic algorithms, may converge to local optima, while the artificial bee colony (ABC) algorithm has better performance in global optimal solution by comparison [49]. Using the ABC algorithm as the benchmark scheme, we can observe that our beamforming algorithm exhibits a performance equivalent to the ABC algorithm with lower calculation complexity.

**Figure 5:**
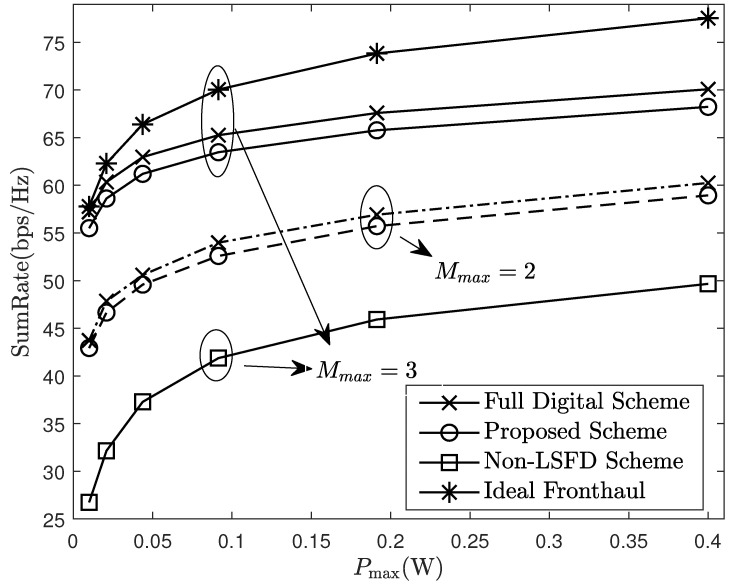
Sum-rate of system under different parameters.

Figure 5 compares the performance under different parameters. It can be observed that sum-rate significantly improves when $M_{max}$ changes from 2 to 3, this is because there are more antennas and RF links to serve users, which improves the diversity gain. Meanwhile, there are also more APs collaborating to decode the uplink data and eliminate the interference between users. Furthermore, Figure 5 verifies the advantage of using the LSFD method at the CPU, which optimizes the LSFD coefficients based on statistical CSI and then combines the weighted signals from different APs. Obviously, compared to simple average combining at the CPU, using the LSFD method can considerably enhance the system performance. From Figure 5, it can also be shown that the performance gap between the hybrid beamforming architecture and full digital architecture is very small. This indicates that the proposed scheme in this paper can greatly reduce the number of RF links without causing significant performance loss, which is beneficial for improving the energy efficiency of the system. Furthermore, compared to the ideal case where the fronthaul capacity is infinite, the actual system performance increases slowly with power growth. This is because as the transmit power increases, the quantization error caused by fronthaul compression becomes more severe, seriously affecting the system performance.

**Figure 6:**
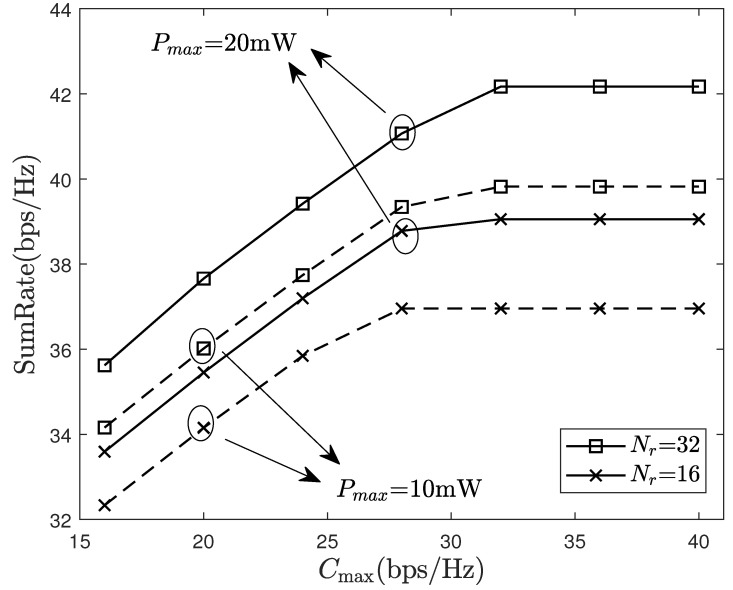
Sum-rate of system with different maximum fronthaul capacity.

Figure 6 compares the system performance under a different maximum fronthaul capacity. Clearly, the system’s sum-rate initially increases and then stabilizes as the fronthaul capacity grows. The reason is that as the fronthaul capacity increases, the AP can more accurately quantify the signal, reducing the signal distortion caused by quantization errors. However, in practice, there is no infinitesimal quantization accuracy, so when the fronthaul capacity is large enough, it will no longer affect system performance. Although increasing the AP antennas offers limited benefits on small capacity fronthaul links, it can significantly improve system performance with large capacity fronthaul links. This is because low capacity fronthaul links introduce significant quantization noise. Note that if the fronthaul rate exceeds the maximum fronthaul capacity, it will not be possible to guarantee the existence of a codebook for error free transmission. In cell-free systems, the fronthaul capacity limitation has an impact on the number of AP antennas, estimating channels, etc. This paper only intends to propose a resource allocation algorithm under the limitation of fronthaul capacity, and the related trade-off issues will be left for future research. Furthermore, in Figure 6, the sum-rate under $N_{r}=16$, $P_{max}=20$ mW is less than that under $N_{r}=32$, $P_{max}=10$ mW, which suggests that we can lower the transmit power at the terminals without reducing the sum-rate by appropriately increasing the antennas at each AP.

**Figure 7:**
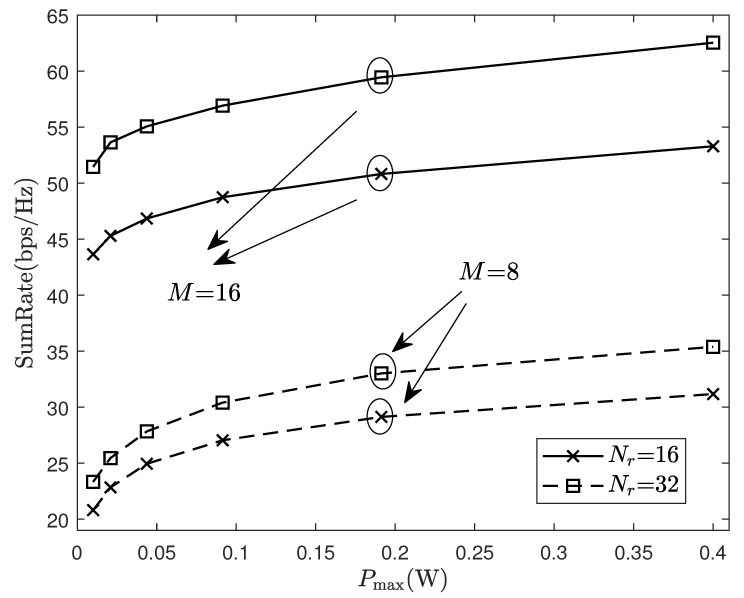
Sum-rate of system with different numbers of APs and antennae.

Figure 7 evaluates the system performance under different numbers of AP and antenna. Assuming that APs randomly spread throughout the region, it is obvious that increasing APs to improve the macro diversity gain, or increasing the antennas at each AP to improve the micro diversity gain, both can improve system performance. Furthermore, the sum-rate under $M\times N_{r}=16\times 16=256$, $N_{RF}=64$ is greater than that under $M\times N_{r}=8\times 32=256$, $N_{RF}=64$, which means that in some cases, the macro diversity has a greater impact on sum-rate than the micro diversity in the system.

**Figure 8:**
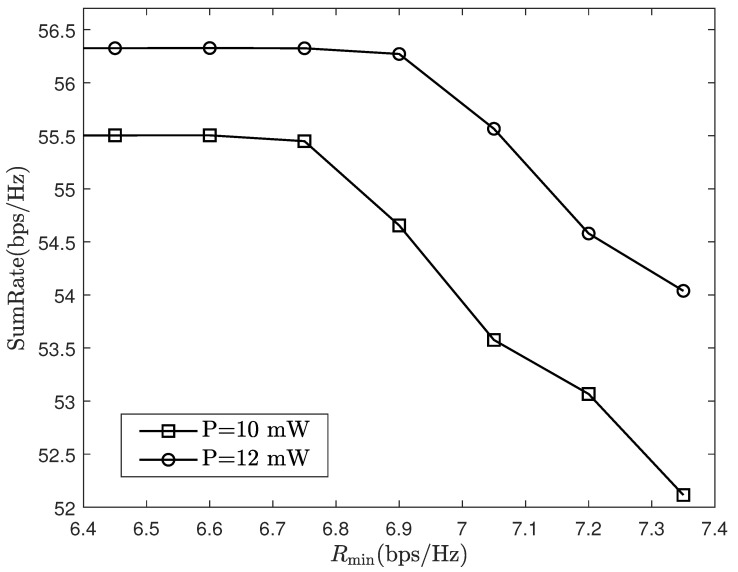
Sum-rate of system under different minimum rate.

Figure 8 depicts how the sum-rate changes in relation to the minimum rate. As the minimum rate increases, the sum-rate is not initially affected. This is because in this instance, the rates in the system are all greater than the minimum rate, and the resource allocation algorithm is not affected by the minimum rate constraint. However, when the minimum rate increases to a certain value, the sum-rate will decrease as it increases. This is because users with poor channels will not be able to obtain the minimum rate under the previous resource allocation scheme. Thus, it is crucial to lower the transmit power of users with good channels to reduce interference to the former, sacrificing the performance of users with good channels to meet the minimum rate indicator that will cause a reduced system sum-rate performance. Unfortunately, the problem of uplink signal synchronization in multi-AP collaborative communication remains to be solved, which will be explored in future research.

## 5. Conclusions

The sum-rate maximization problem is addressed in this paper based on statistical CSI for the cell-free mmWave MIMO system. An AP–user association scheme is first proposed, and then a resource allocation algorithm is proposed based on AO, fractional programming, and the MM method, which jointly optimize the users’ transmit power, quantization error variance and HBF, and the LSFD coefficients to maximize the sum-rate of the system. The experimental findings indicate that the developed strategy yields a sum-rate comparable to that of the benchmark scheme, and using the LSFD method in the CPU can lead to substantial enhancements in system performance. The future work will concentrate on more practical issues such as hardware impairments, uplink synchronization challenges, and broadband communication technology, in order to tackle real-world challenges that may arise.

## Figures and Tables

**Table 2:** Summary of default simulation parameters.

Parameter	Value
Noise power: $\sigma^{2}$	−94dBm [20]
Number of antennas: $N_{r}$	32 [33,47]
Number of RF links: $N_{RF}$	8
Number of users: *K*	6
Carrier frequency: $f_{0}$	73GHz [32]
Number of APs: *M*	16
Maximum transmit power: $P_{max}$	10mW
Minimum rate: $R_{min}$	1bps/Hz
Maximum fronthaul capacity: $C_{max}$	24bps/Hz [21,22]
Number of APs serving each user: $M_{max}$	3
Side of the coverage area: *D*	400 m [46,48]

## Data Availability

Interested parties may obtain the study’s data directly from the corresponding author.
